# Introduction and validation of the open symptom framework: a public domain modular framework for patient-reported measurement of symptoms related to cancer and its treatment

**DOI:** 10.1007/s11136-024-03656-6

**Published:** 2024-07-18

**Authors:** C. Gibbons, G. Brown, S. C. Lu, A. Elrick, Y. Tang, M. Kaufman, M. Williams, C. Xu, C. Harrison, C. Swisher

**Affiliations:** 1 Section of Patient Centered Analytics, Division of Internal Medicine, The University of Texas MD Anderson Cancer Center, 6565, MD Anderson Blvd, Houston, TX 77030 USA; 2 Department of Symptom Research, The University of Texas MD Anderson Cancer Center, Houston, TX USA; 3 The Ronin Project Inc., San Mateo, CA USA; 4 Department of Bioinformatics, University of Texas, El Paso, TX USA; 5 Nuffield Department of Orthopaedics, Rheumatology and Musculoskeletal Sciences, University of Oxford, Headington, Oxford, UK; 6 Ellison Institute of Transformative Medicine, Los Angeles, CA USA

**Keywords:** Patient-reported outcome measure, Open symptom framework, Patient-reported outcome version of common terminology criteria for adverse events, Item response theory

## Abstract

**Purpose:**

We provide an initial description and validation of some public domain patient-reported outcome (PRO) items to assess cancer symptom burden to address immediate barriers to symptom assessment use in clinical practice and facilitate future research.

**Methods:**

We created the Open Symptom Framework (OSF), a flexible tool for clinical cancer-related symptom assessment. The items comprise six components: recall period, concept, symptom, qualifier(s), a definition, and a 5-point Likert-type response. We recruited patients receiving cancer therapy in the United States and United Kingdom. We assessed external construct validity by comparing OSF scores to the PRO-CTCAE measure and assessed reliability, scalability, dimensionality, and item ordering within a non-parametric item response theory framework. We tested differential item functioning for country, age, gender, and level of education.

**Results:**

We developed a framework alongside clinical and psychometric experts and debrieifed with 10 patients. For validation, we recruited 331patients. All items correlated with the PRO-CTCAE equivalents (*r* = 0.55–0.96, all *p* < 0.01). Mokken analysis confirmed the scalability and unidimensionality of all symptom scales with multiple items at the scale (*H*o = 0.61–0.75) and item level (*Hi* = 0.60–0.76). Items are interpreted consistently between demographic groups (*Crit* = 0 for all groups)*.*

**Conclusion:**

The public domain OSF has excellent psychometric properties including face, content, and criterion validity and can facilitate the development of flexible, robust measurements to fulfil stakeholder need. The OSF was designed specifically to support clinical assessment but will function well for research. Further work is planned to increase the number of symptoms and number of questions per symptom within the framework.

## Plain English Summary

Clinical assessment and feedback of cancer-related symptoms using remote patient-reported outcomes (PROs) can improve key patient outcomes including quality of life and survival. More research is needed to fully understand how to optimize remote PRO assessment to maximize actionable information and reduce patient burden. We hope to increase the adoption and innovation surrounding remote PRO symptom assessment by providing a widely available open-source patient-reported outcome measure (PROM). In this study, we provide evidence that Open Symptom Framework is suitable for measuring nine different cancer-related symptoms by comparing it to another commonly used assessment tool as well as showing that the questions are understood in the same way by different people.

## Introduction

Patient-reported outcome measures (PROMs) are tools which can be used to assess patient health and wellbeing directly and without interpretation from a third-party. Data from PROMs have been used for research, auditing, to support labelling claims, and to inform clinical practice [1]. Evidence demonstrates that the use of PROMs to measure and feedback patient-reported outcomes (PROs) in clinical practice may have substantial positive impacts on both cancer care and treatment outcomes without increasing the length of the clinical encounter [2, 3]. For cancer patients receiving systemic therapies, the use of PRO feedback can improve health-related quality of life, mental functioning, patient-provider communication, and 1-year overall survival in advanced cancers [4]. Outside of oncology, PRO feedback interventions have been shown to increase communication, diagnosis and notations, disease control, as well as quality of life [5].

Despite the demonstrated potential of PRO feedback as a clinical intervention uptake across cancer services has been slow [6, 7]. There are many potential barriers to the implementation of these tools which can include lack of stakeholder engagement, cost, and technology barriers. Currently, available instruments may also create barriers to clinical implementation through their licensing agreements. As far as we are aware, there is currently no validated tool to assess symptoms relating to cancer therapy which is both freely available and designed specifically for assessment in clinical practice.

One strategy which has been used to democratize and transform person-centred assessment in other fields, including personality and cognitive assessment, is the creation of public-domain assessments. Public domain assessments are freely accessible for any use and can generally be modified in accordance with their licenses. Both the International Personality Item Pool (IPIP) and the International Cognitive Ability Resource (ICAR) were created with the goal of making psychometric measurement tools easier to access in order to do research to advance understanding of these constructs and how to best measure them in the future [8, 9]. Utilizing an open approach led to continuous refinement from a wide range of stakeholders including, but importantly not limited to, the team who developed the first items and questionnaires in each initiative. The IPIP now contains over 3000 items available in more than 48 languages while the ICAR has around 1,000 ability items in the public domain [9].

However, cancer symptom assessment measures are currently limited by restrictive licenses and data sharing policies, limiting access and continuous refinement and expansion through open collaboration and contribution. These limitations may prohibit the spread and adoption of symptom assessment tool in clinical practice, especially by users who are unable to pay to use symptom assessments, cannot share the data they collect with the assessment developer, or who plan to include the assessment within a tool that will be sold.

Another limitation of current cancer symptom assessment tools is their inability to be modified in any way without express permission of the original Authors. These restrictions create barriers to innovations that could improve PRO assessment. There are many areas that the clinical use of PROMs could benefit from additional research and innovation. For example, strategies to optimize many elements of PROM use, including presentation style and timing of collection and provision of feedback, are not yet clear. Opportunities exist for optimizing which symptoms are asked at what stage of treatment for individuals. Many PROMs were originally developed to support group-based research by providing reliable scores that can be used in statistical operations and facilitate discovery. Though useful for research, the use of a non-standardized PRO score may contribute to the difficulties in implementing PRO feedback into clinical practice as it is not always clear how a certain score on a PROM relates to a potential clinical action. Even if a score is easy to interpret it may still require further contextual information to be actioned. However, due to their research focus, contextual information to guide decision making is not typically included within any currently available cancer symptom assessments.

Unlike PROs that assess one symptom per measure, such as those included in the PROMIS measurement system, many symptom assessment tools utilize an ‘omnibus’ approach that combine multiple cancer-related symptoms into a single measure do not conform to the assumptions of modern psychometric paradigms. The leading paradigm in psychometric assessment is item response theory (IRT) and related approaches such as Rasch analysis. Widely used in all fields that measure intangible abstract phenomena using validated questionnaires, IRT is the gold standard for ensuring measurement precision and well as flexibility. In medicine, IRT is identified and recommended for use in PRO development by the USA Food and Drug Administration [10]. However, applying IRT to omnibus cancer symptom measures has historically proven difficult. Cancer symptoms measures tend to contain multiple symptoms, up to 78 within the PRO-CTCAE, and as such often to rely on a small number of items to measure each individual symptom. Some scales suggest an overall’symptom burden’ score but the principal assumption of IRT is that all items within a questionnaire are unidimensional (i.e., only measure a single construct) and have a consistent probabilistic relationship with one another (i.e., scores are not influenced by external factors).

The three key goals of the OSF are: to increase access to a validated omnibus cancer symptoms assessment through open-source licensing, to simplify the process of item bank expansion and translation though the use of a structured framework, and to provide a tool to improve the use and usefulness of clinical PRO assessment but provide a flexible tool that can support the evaluation of cutting-edge data science techniques.

In this paper, we sought to provide an initial description and validation of some public domain PRO items to assess cancer symptom burden which are designed both to address immediate barriers to the optimal use of symptom assessment in clinical practice as well as to facilitate future research to advance the practice. We set out to demonstrate their validity and suitability for further development into item banks suitable for parametric IRT calibration.

The Open Symptom Framework will be distributed under a Creative Commons CC-BY-SA license, which allows free use, modification, and distribution with proper attribution. Accompanying information relating to the items, scoring, and license are available at opensymptomframework.com. Due to the technical nature of PROM development and validation, the OSF authors and collaborators will not automatically endorse modifications or extensions of the OSF. However, we will regularly review efforts to improve or expand the OSF and maintain a list of endorsed versions on our website.

## Methods

### Population

We recruited cancer patients and survivors though two online web panels Qualtrics and Prolific in the United States of America (USA) and United Kingdom (UK). Participants over the age of 18 were eligible to join the study if they were currently taking cancer therapy or had completed their therapy in the past months. Participants were compensated for their time in accordance with the recommendations on each platform.

### Ronin open symptom framework design

**Fig. 1:**
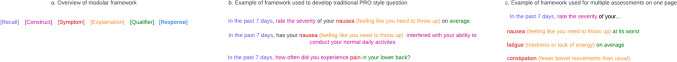
Examples shown of how the OSF framework (panel 1**a**.) can be used to develop and codify PRO questions in both a traditional (panel 1**b**.) and novel format (panel 1**c**.)

The initial development of the Open Symptom Framework (OSF) began with a series of discussions and interviews with clinicians, and psychometricians as well as reviews of the literature. A framework was developed to reflect the common elements of questions in PROMs and assembled each item using elements of the framework. We debriefed the framework using cognitive interviews with 10 cancer patients. The six elements of the framework are the recall period which patients are asked to response in relation to, the conceptual element that is being measures (e.g., severity, frequency, control), the symptom that is being assessed, an optional explanation of that symptom to increase comprehensibility and reduce variation in responses, an optional qualifier (e.g., the location on the body or whether a patient should report the average or worst severity, for example) as well as a Likert-type response options (Fig. 1).

The framework was designed to be scalable and to eventually be able to measure all relevant symptoms. Part of the rationale for developing a framework with discrete elements was that it may be easier to develop new items, to create new language versions, and to interface with natural language processing tools. For the initial validation, we chose nine symptoms that were related to potentially avoidable admissions to the emergency department. Those symptoms were anxiety, constipation, diarrhoea, fatigue, nausea, pain, shortness of breath, vomiting, and fever [11–14].

We designed the OSF to provide measurement at the level of the individual symptom rather than create an aggregate measure of ‘overall symptom burden’ due to the complex multidimensional nature of that construct. We included multiple questions for anxiety, fatigue, nausea, pain, shortness of breath, and vomiting and it is our intention that each of these symptoms will eventually be linked to novel or existing item banks (e.g., PROMIS) to create more comprehensive measurement of each construct should it be required. When expanded beyond 2–3 questions we hope to fit the items in each symptom to a parametric item response theory model to create item parameters facilitate computerized adaptive testing and other algorithmic administration techniques. In total, the nascent OSF used in this study contained 18 questions of which 15 had an equivalent in the PRO-CTCAE to serve as a comparison for this validation study.

We used 5-point Likert response plus an additional binary screening question which was, by default, set to a negative response (i.e., no symptom present). The rationale for screening question was to simultaneously minimize assessment burden while providing the option to respond to all relevant symptoms. We consider this technique to be the most scalable to facilitate the assessment a greater number of symptoms in the future without unduly increasing patient burden [15].

### Contextual questions

We have included contextual questions within the OSF which are designed to enhance the interpretation of PRO scores. These included questions such as “is this symptom a new problem for you?” and “have you taken medication for this symptom?”. Because these questions are designed to provide context, their responses are not expected to be  ordinally scaled and do not represent a consistent underlying construct, as such they were omitted from psychometric analysis and will not contribute to symptom severity scores. Contextual questions were added for all symptoms, and we rely solely on thermometer readings and thus classified all questions relating to fever as contextual. Full details of the clinical contextual questions are provided in Supplementary Materials 1.

### Psychometric evaluation

We recruited patients using the Qualtrics and Prolific recruitment platforms [16, 17]. Details of the recruited sample can be found in Table 1. We determined a sample of 300 patients to have sufficient information to conduct Mokken analysis and be sufficiently powered for statistical comparisons between the nascent OSF and the gold-standard PRO-CTCAE.

**Table 1:** Demographic information and clinical characteristics for survey participants

Variables	Classification	Mean/count	SD/percentage
Age		42.0	14.0
Gender	Female	145	43.8%
	Male	182	55.0%
	Other	1	0.4%
Ethnicity	Hispanic, Spanish, or Latino	95	28.8%
	Non-Hispanic, Spanish, or Latino	235	71.2%
Education	Some high school or less	5	1.5%
	High school diploma or GED	39	11.8%
	Some college, but no degree	48	14.5%
	Associates or technical degree	58	17.6%
	Bachelor’s degree	92	27.9%
	Graduate or professional degree (MA, MS, MBA, PhD, JD, MD, DDS. Etc.)	88	26.7%
Employment	Unemployed	18	5.5%
	Student	8	2.4%
	Working part time	25	7.6%
	Homemaker or stay-at-home parent	12	3.6%
	Working full-time	221	67.0%
	Retired	40	12.1%
	Other	6	1.8%
Marital status	Single	51	15.5%
	Divorced	17	5.2%
	Widowed	13	3.9%
	Living with a partner	16	4.8%
	Married	233	70.6%
Income	Less than $25,000	24	7.3%
	$25,000 to $49,999	47	14.2%
	$50,000 to $74,999	51	15.5%
	$75,000 to $99,999	57	17.3%
	$100,000 to $149,999	115	34.8%
	$150,000 or more	36	10.9%
Treatment (current and historic)	Chemotherapy	213	64.5%
	Radiation	139	42.1%
	Immunotherapy	139	42.1%
	Surgery	144	43.6%
	Targeted therapy	59	17.9%
	Hormone therapy	108	32.7%
	Other oral therapy	136	41.2%
Cancer type	Bladder	19	5.8%
	Breast	68	20.6%
	Colon and rectum	23	7.0%
	Head and neck cancer	1	0.3%
	Kidney, renal, and pelvis	5	1.5%
	Leukemia	17	5.2%
	Lung and bronchus	32	9.7%
	Melanoma and skin	100	30.3%
	Non-Hodkin Lymphoma	5	1.5%
	Pancreas	5	1.5%
	Prostate	15	4.5%
	Testicular cancer	1	0.3%
	Thyroid	10	3.0%
	Uterus	9	2.7%

*SD* standard deviation, *GED* general educational development

For 15 of the 18 OSF questions, we assessed Spearman’s rank correlation between the OSF symptom questions and their equivalent PRO-CTCAE item.

We assessed ceiling effects by counting the number of patients who scored at the polar extremes of each scale. Floor and ceiling effects up to 20% have been shown to have minimal impact on the validity of the data collected. Due to the intention that the OSF relies on an preliminary screen for symptoms, which we expect will not always be present, floor effects were not assessed as an indicator of psychometric performance. We compared average ceiling effects between the OSF and the PRO-CTCAE but did not perform a statistical evaluation with only 15 comparisons.

We set up our study such that if a patient said they did not experience a particular symptom, they were shown neither the relevant OSF nor PRO-CTCAE questions. As the PRO-CTCAE is designed to work without a separate screening questionnaire, the initial response category is “never”, “none”, or “not at all”. We therefore assessed responses to those categories as an indicator of data quality; expecting the average number of responses in those categories to approach zero. As patients who do not experience a particular symptom at all are sometimes required to respond to multiple questions, we calculated an estimated reduction in burden for each symptom as a result of the single item screener, compared to the number of items that would have been posed in the PRO-CTCAE to gather the same information.

We used Mokken analysis to assess the scalability, dimensionality, and differential item functioning (DIF) of the OSF symptom measure. Mokken analysis is a non-parametric item response theory method. Within Mokken analysis, *H* (Loevinger’s coefficient) measures the extent to which all items are arranged as expected by their mean values along the latent trait. A Loevinger’s coefficient > 0.3 is the minimum acceptable value of *H* indicating a weak scale; *H* > 0.4 indicates a moderate scale; and *H* > 0.5 indicates a strong scale.

To assess whether our nascent questionnaire may be suitable for future 1-parameter ‘Rasch’ IRT modeling, we investigated the assumptions of invariant item ordering (IIO) and non-intersection. We looked at the number of violations and significant violations of these assumptions in our data. We assess internal consistency reliability by calculating *Rho*, an alternative to Cronbach’s alpha statistic developed specifically for use in Mokken analysis [18].

Differential item functioning, or DIF, was assessed to ensure that item ordering is consistent between demographic subgroups. The *crit* value is used to assess ordering whereby a value greater than 80 indicates likely DIF, and a value below 40 suggests no DIF [19]. We assessed DIF between male and female genders, younger (< 40) and older (> 40) patients, those with and without college-level education, and participants from the UK and the US.

We did not assess test–retest validity as we expect that symptoms will change over time and at different rates, either due to medical intervention or natural history.

## Results

In our cognitive debrief, we recruited 10 patients comprising 5 women and 5 men who had been diagnosed with prostate, breast, colorectal, lung, or bone marrow cancers. Participants ranged from 22 to 71 years old (mean 55) and had experience with chemotherapy, surgery, radiation, hormonal therapy, and immunotherapy. We recruited 251 patients with cancer from the United States to complete the nascent questionnaire as well as items from the PRO-CTCAE and several demographic and disease-related questions. Descriptive statistics for the sample are given in Table 1. On average, patients reported 3.99(±1.32) symptoms. The inclusion of a screening stage before multiple item for each symptom resulted in the reduction of an average of 4 items per participant, a 25% reduction in length compared to the PRO-CTCAE.

Correlations between the 15 OSF questions and their PRO-CTCAE equivalents were all significant and typically of high (14/15; *r* > 0.60, *p* < 0.05) with just one comparison being moderate.

Ceiling effects across all individual items of the OSF were, on average 9.12 ± 3% (SD 3%) which was slightly lower than the equivalent PRO-CTCAE questions (10.8 ± 3.2%). As expected, floor effects for the PRO-CTCAE were minimal, with an average of 0.86 ± 0.78% of scores in the lowest category per item.

The AISP Mokken analysis confirmed that each of the six scales comprising two or more items produced measurement along a single dimension. Our assessments indicated that all items and scales demonstrate strong scalability (Loevinger’s *H* > 0.50; see Table 2). Reliability was acceptable for all scales (Rho 0.76–0.81).

**Table 2:** Psychometric properties for open symptom framework questionnaire demonstrating reliability, strong scalability, and no violations of the IIO scales

Symptom	Item wording	Response	Item Score^a^					Ccorrelation with PRO-CTCAE	Item scalability	Overall scalability	*Reliability-Rho value*
		*n* (%)	1	2	3	4	5	r^b^	H(se)	H(se)	
Anxiety	In the past 7 days, how often did you experience anxiety?	203(61.3)	6	47	79	55	16	0.64***	0.60(0.08)	0.61(0.06)	0.81
	Rate the severity of your anxiety in the past 7 days at its worst	203(61.3)	7	27	82	63	24	0.71***	0.68(0.06)		
	In the past 7 days, has your anxiety interfered with your ability to do your normal daily activities?	203(61.3)	6	43	81	58	15	0.73***	0.65(0.06)		
Constipation	Rate the severity of your constipation (fewer bowel movements than usual) in the past 7 days at its worst	140(42.3)	9	23	36	59	13	0.67***	NA	NA	NA
Diarrhoea	In the past 7 days, how often did you experience diarrhoea (loose or watery stool)?	156(47.1)	6	33	57	49	11	0.67***	NA	NA	NA
Fatigue	Rate the severity of your fatigue (tiredness or lack of energy) in the past 7 days at its worst	240(72.5)	3	25	89	95	28	0.96***	0.75(0.03)	0.75(0.03)	0.82
	In the past 7 days, has your fatigue (tiredness or lack of energy) interfered with your ability to do your normal daily activities?	240(72.5)	4	44	78	95	19	0.94***	0.75(0.03)		
Nausea	In the past 7 days, how often did you experience nausea (feeling like you need to throw up)?	155(46.8)	9	31	43	59	13	0.55***	0.70(0.04)	0.68(0.05)	0.80
	Rate the severity of your nausea (feeling like you need to throw up) in the past 7 days at its worst	155(46.8)	4	20	48	66	17	0.68***	0.66(0.05)		
	In the past 7 days, has your nausea (feeling like you need to throw up) interfered with your ability to do your normal daily activities?	155(46.8)	7	23	42	71	12	NA	0.67(0.05)		
Pain	In the past 7 days, how often did you experience pain?	202(61)	1	16	51	70	9	0.69***	0.74(0.04)	0.74(0.04)	0.83
	In the past 7 days, has your pain interfered with your ability to do your normal daily activities?	202(61)	3	10	60	59	15	0.75***	0.74(0.04)		
Shortness of breath	In the past 7 days, how often did you experience shortness of breath?	119(36)	3	28	41	40	7	NA	0.67(0.07)	0.72(0.05)	0.85
	Rate the severity of your shortness of breath in the past 7 days at its worst	119(36)	3	15	47	48	6	0.60***	0.76(0.05)		
	In the past 7 days, has your shortness of breath interfered with your ability to do your normal daily activities?	119(36)	4	18	39	48	10	0.70***	0.74(0.05)		
Vomiting	In the past 7 days, how often did you experience vomiting (throwing up)?	80(24.2)	2	15	33	27	3	0.63***	0.67(0.07)	0.67(0.06)	0.83
	Rate the severity of your vomiting (throwing up) in the past 7 days at its worst	80(24.2)	2	10	28	32	8	0.81***	0.73(0.06)		
	In the past 7 days, has your vomiting (throwing up) interfered with your ability to do your normal daily activities?	80(24.2)	4	10	31	32	3	NA	0.67(0.06)		

^a^Item score 1 = (rarely, mild, not at all); Item score 2 = (sometimes, mild, a little bit); Item score 3 = (often, moderately, somewhat); Item score 4 = (most of the time, severe, very much); Item score 5 = (all the time, very severe, an extreme amount)

^b^*p < 0.05; **p < 0.01; ***p < 0.001

There were no violations of the assumption of IIO for the scales using the overall data (Table 2), or for each of the demographic subgroups (all comparisons Crit value = 0; Table 2).

## Discussion

In this paper, we articulate an open-source framework for assessing symptoms relating to cancer and its treatment. The initial items of the OSF demonstrate convergent validity with existing symptom assessments and have good scalability, unidimensionality, and function consistently across demographic groups. Additionally, we demonstrate some benefits to the OSF compared to the benchmark measure in terms of a marginally reduced ceiling effect and a substantial reduction in the average number of items included in the assessment. To our knowledge, the OSF is the first public domain cancer symptom assessment that was developed specifically to support symptom monitoring in clinical practice. It is also, to our knowledge, the first PRO assessment tool which is explicitly based on an explicit modular framework. The OSF is distributed under a Creative Commons CC-BY-SA license and we enthusiatically invite others to contribute to the development and refinement of the OSF. We beleive that theframework that can benefit from the input of healthcare professionals, researchers, and patients alike. Additionally, we will release “official parameters” for the use of the OSF and will provide guidelines on how to administer and interpret the assessment results in clinical practice on our website https://www.opensymptomframework.com/.

The historic absence of a public-domain measure which has been designed specifically for remote assessment and communication of cancer symptoms during therapy may have been a barrier to the adoption of these tools in clinical practice. Many other cancer symptom measures are suitable for use in clinical practice, but, to our knowledge, all other measures were developed and optimized for use in research and especially clinical trials. We are encouraged to see the success of public domain assessment initiatives in both personality and cognitive assessment in the form of the IPIP and the ICAR. In both instances, researchers have increased access to measures of these important domains and enabled the research to drive the science of assessment in these areas [8, 20].

We are excited about the new possibilities and efficiencies that the modular framework could bring to the science of assessing patient symptoms. Previously, it was usually understood that questions within a PRO could only be considered valid if they were reproduced in a form, and sometimes format, which matched the original exactly. We hope that experiments with the framework will examine whether validity exists at the semantic level (*i.e.,* valid if the *meaning* of the question is unchanged) or, as has previously been claimed, at the literal level (*i.e.,* no element of the question can be altered). Understanding the limits of validity could reduce research waste and inform new types of assessment using, for example, natural language or chatbots. Chatbots may be a useful tool for soliciting responses from patients because they could promote patient engagement by providing a more interactive experience, which encourages patients to provide more detailed responses [21, 22]. We note that such research would be impossible with current restrictions on the use of current omnibus symptom measures. The flexibility provided by our modular framework could support research which may lead to psychometrically calibrated chatbots that were able to ask a variety of standardized questions while incorporating context specific to the patient.

The structured framework within the OSF could facilitate the addition of more symptomsand potentially provide greater rigor in qualitative evaluation of new item content. We expect that elements of the framework, once validated, can be duplicated to assist with the development of new items. For example, if the framework construct of “interference with activities of daily living” was shown to perform well psychometrically for one symptom where it was chosen by patients to be relevant, then it may be used for another new symptom where patients had suggested it was also relevant. The repetition of common elements of the framework across symptoms may create new opportunities to compare the relative impact of individual symptoms or symptom clusters.

In the current study, we chose to validate items that have substantial conceptual overlap with the benchmark PRO-CTCAE measure to establish the validity of these core items which we intend to expand upon in future research. The measurement concepts which are explicitly present in both the OSF and the PRO-CTCAE, such as severity, impairment, and frequency, are also present in many other PROs that assess symptoms, such as the PROMIS Fatigue or Pain item banks.

The six components of the symptom OSF may also facilitate the development of new techniques to assess patient symptoms. These components could be used like labels generated in the annotation of open text used in natural language processing studies [23]. We expect that the OSF could be used as annotation labels to facilitate the cross-calibration of PRO questionnaire scores and patient symptom information taken from other sources, such as clinical narratives, documented telephone encounters, or information collected using chatbots or posted on the internet, for automatically generate new items. In addition, it could be used to accelerate the labeling of key events in data derived from remote patient monitoring devices and wearables (e.g., activity trackers and continuous monitoring).

Future research could expand the OSF either to capture other relevant concepts such as social interference, ability to control, or time spent thinking within its own framework or to psychometrically calibrate the core items of the OSF alongside established measures such as PROMIS to facilitate metric comparisons. We believe there is significant opportunity to accelerate cancer research efforts around the globe by leveraging the translation facilities provided by modern linguistic agents, such are large language models (LLMs). There is substantial work in this area to validate and assess the costs and benefits of agent-based translations compared to human translations. Regardless of translation methodology, we would like to impress the importance of ensuring cultural, as well as linguistic, equivalency for each of the item developed in the future.

A key goal for the OSF project is to reduce, wherever possible, the burden on researchers to develop or validate new elements of the framework and to increase clinical impact at scale via openness to use by commercial and non-commercial entities alike. We hope that the OSF can be used to create research findings that are broadly applicable for cancer symptom measurement generally, and thus reduce replication of studies and the creation of knowledge which is limited to a single tool. For example, the MDASI has been assessing symptom severity using a one-day recall for over 20 years [24], and recent research has been conducted with 118 cancer patients to demonstrate that the PRO-CTCAE is also suitable to assess many of the same symptoms also using a one-day recall period [25].

There are some limitations to the current study. Although we were able to demonstrate a few advantages of the OSF compared to the benchmark measure, there is work still to be done to validate the clinical utility of the scores as they relate to urgent clinical needs. We are aware that false positive clinical alerts arising from symptom assessments are problematic and create a significant barrier to their widespread uptake. In our previous work, we used smart rules-based alerts to reduce alert fatigue for providers by a 33% improvement in positive predictive value with 18% of unnecessary symptom alerts avoided and a 0% decrease in sensitivity [26]. Future research may use data science techniques to ensure that clinical alerts arising from PROs are optimally tuned to reduce false positives and negatives.

The sample which we recruited was, on average, younger than a general population cancer sample which was likely a reflection of the web panel recruitment, especially in the sample recruited from Qualtrics in the United States. Despite this caveat, our DIF analyses did suggest that items were understood in the same way between younger and older patients within our sample which is consistent with our assumption that the content of our assessments is relevant to all adults. We note that, depending on the goal of the research, samples which closely represent the population in their distribution of key elements like age, gender, and sociodemographic status may not be necessary. We are satisfied that the sample we recruited was sufficiently large and diverse for the purposes of this investigation but would recommend that future studies that seek, for example, to create parameters using parametric IRT models or to characterize the symptom burden experienced by a particular patient group seek to recruit samples that are more representative of the specific groups they are interested in, though some argue this is still not necessary if sample independent Rasch models are utilized [27].

We hope that modern techniques and technologies might be used to reduce the research effort needed to expand the framework so that efforts can be focussed on generating new knowledge around how to optimize PRO assessment and feedback to maximize patient outcomes. For example, the use of an item anchoring methodology could facilitate the development of new framework elements during routine data collection without the need for a separate research sample. The practical benefits of this method have been enjoyed by psychometricians working in education assessment for many years. While these techniques have been proposed for adoption in oncologic assessment, to our knowledge, they have not yet been implemented.

## Data Availability

Data are available to those wishing to collaborate on research regarding the OSF. Please contact the research team.
