# Correction: Introduction and validation of the open symptom framework: a public domain modular framework for patient-reported measurement of symptoms related to cancer and its treatment

**DOI:** 10.1007/s11136-024-03757-2

**Published:** 2024-08-28

**Authors:** C. Gibbons, G. Brown, S. C. Lu, A. Elrick, Y. Tang, M. Kaufman, M. Williams, C. Xu, C. Harrison, C. Swisher

**Affiliations:** 1 Section of Patient Centered Analytics, Division of Internal Medicine, The University of Texas MD Anderson Cancer Center, 6565, MD Anderson Blvd, Houston, TX 77030 USA; 2 Department of Symptom Research, The University of Texas MD Anderson Cancer Center, Houston, TX USA; 3 The Ronin Project Inc., San Mateo, CA USA; 4 Department of Bioinformatics, University of Texas, El Paso, TX USA; 5 Nuffield Department of Orthopaedics, Rheumatology and Musculoskeletal Sciences, University of Oxford, Headington, Oxford, UK; 6 Ellison Institute of Transformative Medicine, Los Angeles, CA USA

**Correction to: Quality of Life Research**

10.1007/s11136-024-03656-6

In the original published article, the corresponding author name “C. Gibbons” was incorrectly written as “Chris Chris Sidey-Gibbons”.

The original article has been corrected.

